# Cord insertion anomalies and adverse pregnancy outcomes: more than an incidental finding

**DOI:** 10.1590/1806-9282.20251878

**Published:** 2026-06-19

**Authors:** Hasan Süt, Mustafa Koçar, Gülşah Aynaoğlu Yıldız

**Affiliations:** 1 Ankara University, School of Medicine, Department of Obstetrics and Gynaecology – Ankara, Turkey.

**Keywords:** Pregnancy outcomes, Ultrasound, Prenatal diagnosis, Premature birth, Umbilical cord abnormalities, Placenta abnormalities

## Abstract

**OBJECTIVE:**

The aim of this study was to investigate the importance of routine evaluation of cord insertion during the prenatal period. To this end, the perinatal outcomes of marginal and velamentous cord insertions were compared to normal cord insertions.

**METHODS:**

We conducted a retrospective cohort study of singleton pregnancies delivered after 24 weeks between 2016 and 2023, comparing marginal and velamentous cord insertions diagnosed on second-trimester ultrasound with normal cord insertions in terms of preterm birth and adverse perinatal outcomes, after excluding cases with fetal anomalies, placenta previa, placental invasion, vasa previa, or multiple gestations.

**RESULTS:**

Among 6,675 singleton pregnancies, 367 (5.5%) had marginal cord insertion and 45 (0.7%) had velamentous cord insertion. Marginal cord insertion was associated with a nearly threefold higher risk of preterm birth before 37 weeks, while no significant differences were observed for other adverse outcomes. In contrast, velamentous cord insertion was linked to significantly increased risks of preterm birth, primary cesarean delivery, low Apgar scores, and neonatal intensive care unit admission compared with normal cord insertions.

**CONCLUSION:**

Marginal and velamentous cord insertions showed associations with preterm birth and certain adverse perinatal outcomes. Routine evaluation of cord insertion may provide useful information for clinical follow-up.

**Clinical Trial Registration:**

This study was retrospectively registered and authorized by the local Ethics Committee of Ankara University (clinical trial date 19.08.2025; number: 1942038).

## INTRODUCTION

The location of umbilical cord insertion (UCI) into the placenta is typically categorized as central, eccentric, marginal, or velamentous^1^. Among these, central and eccentric insertions are typically considered normal. Marginal cord insertion (MCI) is commonly defined as an insertion point located within 2 cm of the placental edge^2^. Velamentous cord insertion (VCI), on the other hand, occurs when the cord inserts into the fetal membranes instead of the placental disk, leaving the vessels unprotected as they traverse toward the placenta^3^.

The UCI site can influence placental blood flow and fetal development^4,5^. Although several studies and meta-analyses have explored the association between abnormal UCI and adverse pregnancy outcomes such as fetal growth restriction, preterm birth, low Apgar scores, and increased rates of cesarean delivery, inconsistencies remain. This is particularly evident in second-trimester evaluations, as many studies are limited by small sample sizes, heterogeneity in study design, and variability in ultrasound timing and diagnostic criteria^6,7^. While the routine assessment of the UCI is not universally part of mid-trimester anomaly scans, current guidelines recommend reporting marginal or velamentous insertions when they are identified^8^. This emphasizes the ongoing debate and variability in clinical practice.

The present study aimed to evaluate the perinatal and neonatal outcomes associated with marginal and VCIs compared to normal cord insertions. We also sought to determine whether abnormal UCI is an independent risk factor for preterm delivery before 37 weeks of gestation.

## METHODS

This retrospective cohort study was carried out at Ankara University Faculty of Medicine. The Ankara University Ethics Committee granted ethical approval on (12.08.2025) with approval number (1942038). Singleton pregnancies delivered at ≥24 weeks of gestation between February 2016 and December 2023 were reviewed. Pregnancies were excluded if a second-trimester ultrasound report was missing, the placental cord insertion site was not documented, or if structural or genetic fetal anomalies were present. Additional exclusion criteria included placenta previa, placental invasion anomalies, vasa previa, and multiple gestations. Following the application of these criteria, cases meeting the eligibility requirements were included in the analysis.

The placental cord insertion site was assessed using a Voluson E8 ultrasound machine (GE Healthcare, Zipf, Austria). Evaluations were performed by two experienced perinatologists, both with more than 10 years of expertise in fetal ultrasonography. In cases of diagnostic uncertainty, a third expert opinion was obtained. The assessment was conducted in accordance with the guidelines of the International Society of Ultrasound in Obstetrics and Gynecology (ISUOG)^8^. Cord insertion was evaluated using 2D grey-scale imaging and color or power Doppler in both the longitudinal and axial planes. MCI was defined as cord insertion within 2 cm of the placental edge. VCI was defined as the umbilical vessels inserting into the membranes before reaching the placental surface, with no protection from Wharton’s jelly^9^.

The following maternal characteristics were recorded: maternal age, gravidity, nulliparity, presence of comorbidities (hypertension, diabetes, preeclampsia, gestational hypertension, renal diseases, cardiac diseases, hypothyroidism, and hyperthyroidism), gestational age at diagnosis (in weeks), and history of uterine surgery. Delivery-related variables such as gestational age at birth, fetal sex, and mode of delivery were also documented.

The perinatal and neonatal outcomes that were assessed included pre-eclampsia, being small for gestational age (SGA), intrauterine growth restriction (IUGR), preterm birth before 37 or 34 weeks of gestation, stillbirth, emergency cesarean delivery (CD), primary CD, Apgar scores of less than 7 at 1 and 5 min, and admission to the neonatal intensive care unit (NICU). Prenatal outcomes in the marginal and VCI groups were compared with those in the normal cord insertion group. In addition, to identify independent factors associated with preterm birth before 37 weeks of gestation, univariate and multivariate logistic regression analyses were performed including maternal age,maternal comorbidity status, SGA, IUGR, type of cord insertion, CD, and preeclampsia as covariates. Maternal comorbidity was defined as the presence of at least one of the following conditions: hypertension, diabetes mellitus, preeclampsia, gestational hypertension, renal disease, cardiac disease, hypothyroidism, or hyperthyroidism.

### Statistical analysis

All statistical analyses were performed using IBM SPSS Statistics for Windows, Version 26.0 (IBM Corp., Armonk, NY, USA). Continuous variables were assessed for normality using the Kolmogorov-Smirnov test. Data were presented as mean ± standard deviation (SD) for normally distributed variables, and as median (interquartile range) for non-normally distributed variables. Categorical variables were expressed as numbers and percentages.

For comparisons between groups, the chi-square test or Fisher’s exact test was used for categorical variables, and the independent samples t-test or Mann-Whitney U test was applied for continuous variables, as appropriate.

To identify factors associated with preterm birth before 37 weeks of gestation, univariate logistic regression analyses were first performed. Variables with p<0.20 in univariate analysis were included in a multivariate logistic regression model. Results were reported as odds ratios (ORs) with 95%CIs. A p<0.05 was considered statistically significant.

## RESULTS

Between February 2016 and December 2023, data from a total of 7,980 deliveries at our clinic were reviewed. A total of 828 patients were excluded due to the absence of a second-trimester ultrasound or undocumented cord insertion site. Additionally, 84 cases with structural or genetic anomalies, 38 cases with placenta previa or placental invasion abnormalities, four cases with vasa previa, and 425 multiple pregnancies were excluded. Ultimately, 6,675 singleton pregnancies were included in the final analysis. Of these, 367 cases (5.5%) had MCI, 45 cases (0.7%) had VCI, and the remaining 6,263 cases (93.8%) had normal cord insertion.

The marginal and normal cord insertion groups exhibited similar characteristics regarding gravidity, nulliparity, gestational age at diagnosis, fetal gender, and mode of delivery. However, maternal age (p<0.001) and the presence of comorbidities (p<0.001) were significantly higher in the MCI group. Additionally, gestational age at delivery (p<0.001) and birthweight (p=0.002) were significantly lower in this group (Table 1).

**Table 1:** Demographic features of marginal and normal cord insertion groups.

	Marginal cord insertion n=367	Normal cord insertion n=6,263	p-value
Maternal age (y)	29 (18–45)	28 (18–49)	**<0.001**
Gravidy	2 (1–8)	2 (1–10)	0.063
Nulliparity	132 (36%)	2,324 (37.1%)	.67
Comorbidity	203 (55.3%)	1,532 (24.5%)	**<0.001**
GA at diagnosis	21 (17–25)	21 (17–25)	0.092
The history of uterine surgery	90 (24.5%)	1,503 (24%)	0.80
GA at delivery (w)	38 (29–42)	39 (24–42)	**<0.001**
Gender (female)	176 (48.2%)	3,019 (48.2%)	0.94
Cesarean delivery	195 (53.1%)	3,025 (48.3%)	0.72
Birthweight (g)	3,190 (925–5,000)	3,250 (330–5,340)	**0.007**

The data are presented as median (minimum–maximum) or number (percentage). Statistically significant values (p<0.05) are in bold. GA: gestational age; g: gram; w: weeks; y: years.

In the comparison of perinatal and neonatal outcomes between the marginal and normal cord insertion groups, no significant differences were observed in the rates of preeclampsia, IUGR, SGA, stillbirth, emergency CD, low 1- and 5-min Apgar scores, or NICU admission. However, the MCI group had significantly higher rates of preterm birth before 37 weeks (OR 2.99; 95%CI 2.29–3.90; p<0.001) and primary CD (OR 1.31; 95%CI 1.02–1.68; p=0.032) (Table 2).

**Table 2:** Perinatal and neonatal outcomes in marginal and normal cord insertion groups.

	Marginal cord insertion n=367	Normal cord insertion n=6,263	OR	95%Cl	p-value
Preeclampsia	7 (1.9%)	161 (2.6%)	0.73	0.34–1.57	0.42
SGA	56 (15.3%)	789 (12.6%)	1.2	0.83–1.74	0.32
IUGR	23 (6.3%)	306 (4.9%)	1.1	0.63–1.9	0.73
Preterm delivery (<37 w)	78 (21.3%)	518 (8.3 %)	2.99	2.29–3.90	**<0.001**
Preterm delivery (<34 w)	5 (1.4%)	123 (2%)	0.68	0.28–1.69	0.42
Stillbirth	2 (0.5%)	18 (0.3%)	1.9	0.43–8.22	0.39
Emergency CD	15 (4.1%)	256 (4.1%)	1.0	0.58–1.7	0.99
Primary CD	108/276 (39.1%)	1,559/4,743 (32.9%)	1.31	1.02–1.68	**0.032**
1-min Apgar score of <7	15 (4.1%)	298 (4.8%)	0.71	0.38–1.31	0.28
5-min Apgar score of <7	4 (1.1%)	36 (0.6%)	2.64	0.8–8.75	0.11
NICU admission	18 (4.9%)	195 (3.1%)	1.6	0.98–2.64	0.059

The data are presented as number (percentage). Statistically significant values (p<0.05) are in bold. CD: cesarean delivery; CI: confidence interval; IUGR: intrauterine fetal growth restriction; NICU: neonatal intensive care unit; OR: odds ratio; SGA: small for gestational age.

No significant differences were observed in the rates of preeclampsia, IUGR, SGA, stillbirth, or emergency CD when the perinatal outcomes of the 45 cases with VCI were compared to those with normal cord insertion. However, the VCI group had significantly higher rates of preterm birth <37 weeks (OR 3.58; 95%CI 1.8–7.1; p<0.001) and <34 weeks (OR 7.68; 95%CI 3.2–18.4; p<0.001), as well as higher rates of primary CD (OR 42; 95%CI 1.24–4.72; p=0.009), low 1-min (OR 3.67; 95%CI 1.62–8.3; p=0.002) and 5-min (OR 16.8; 95%CI 5.72–49.4; p<0.001) Apgar scores, as well as NICU admissions (OR 5.71; 95%CI 2.52–12.9; p<0.001) (Table 3).

**Table 3:** Perinatal and neonatal outcomes in velamentous and normal cord insertion groups.

	Velamentous cord insertion n=45	Normal cord insertion n=6,263	OR	95%Cl	p-value
Preeclampsia	3 (6.7%)	161 (2.6%)	2.7	0.82–8.8	0.10
SGA	8 (17.8%)	789 (12.6%)	1.5	0.7–3.2	0.30
IUGR	4 (8.9%)	306 (4.9%)	1.9	0.67–5.32	0.22
Preterm delivery (<37 w)	11 (24.4%)	518 (8.3 %)	3.58	1.8–7.1	**<0.001**
Preterm delivery (<34 w)	6 (13.3%)	123 (2%)	7.68	3.2–18.4	**<0.001**
Stillbirth	0	18 (0.3%)	0	0	0.99
Emergency CD	4 (8.9%)	256 (4.1%)	2.28	0.81–6.42	0.11
Primary CD	19/35 (54.3%)	1,559/4,743 (32.9%)	2.42	1.24–4.72	**0.009**
1-min Apgar score of <7	7 (15.6)	298 (4.8%)	3.67	1.62–8.3	**0.002**
5-min Apgar score of <7	4 (8.9%)	36 (0.6%)	16.8	5.72–49.4	**<0.001**
NICU admission	7 (15.6%)	195 (3.1%)	5.71	2.52–12.9	**<0.001**

The data are presented as number (percentage). Statistically significant values (p<0.05) are in bold. CD: cesarean delivery; CI: confidence interval; IUGR: intrauterine fetal growth restriction; NICU: neonatal intensive care unit; OR: odds ratio; SGA: small for gestational age.

To further evaluate the increased rate of preterm birth (<37 weeks) observed in both the MCI and VCI groups compared to the normal cord groups, a multivariable regression analysis was performed. Both MCI (OR 2.8; 95%CI 2.1–3.8; p<0.001) and VCI (OR 3.1; 95%CI 1.5–6.3; p=0.002) remained significant independent risk factors for preterm delivery in the adjusted model. In addition, preeclampsia, IUGR, CD, and the presence of maternal comorbidities were also independently associated with an increased risk of preterm birth (p<0.001, p<0.001, p<0.001, and p=0.013, respectively).

## DISCUSSION

In this study, MCI was not linked to significant differences in adverse perinatal or neonatal outcomes such as preeclampsia, IUGR, SGA, stillbirth, emergency cesarean, low Apgar scores, or NICU admission. However, MCI was associated with a significantly higher risk of preterm birth before 37 weeks, occurring almost three times more often compared with normal cord insertion. In contrast, VCI was linked to markedly increased adverse outcomes, including higher rates of preterm birth (<37 and <34 weeks), low Apgar scores, and NICU admissions. Overall, these findings suggest that assessing cord insertion may be justified as part of routine obstetric evaluation, considering its potential impact on pregnancy outcomes.

A meta-analysis investigating risk factors for MCI identified chronic hypertension, placenta previa, and nulliparity as significant contributors^10^. However, our study found that nulliparity was not a significant risk factor, whereas advanced maternal age and maternal comorbidities were.

Several retrospective studies have assessed the impact of MCI on perinatal outcomes. A 2021 study with 183 cases found no significant differences in intrapartum fetal intolerance, Apgar scores, birth weights, delivery complications, or NICU admissions^6^. Additionally, a 2022 study with 76 MCI cases (4.2%) and a 2025 study with 25 cases (5.6%) reported no statistically significant differences in adverse perinatal outcomes^1,11^. Conversely, a meta-analysis published in 2023, which combined data from 15 studies, found that MCI was associated with a higher risk of SGA, preeclampsia, stillbirth, preterm birth, and low birth weight^7^. Consistent with these results, the prevalence of MCI in this study was 5.5% (n=367), which is in line with previously reported rates. Notably, the MCI group showed a significantly increased risk of preterm birth and primary CD, while no significant differences were seen in other adverse perinatal or neonatal outcomes.

VCI has been more consistently associated with adverse perinatal outcomes compared to MCI, as shown in several studies^1,12,13^. A meta-analysis published in 2023 reported an overall incidence of 1.4% for VCI and demonstrated increased risks of SGA, preterm birth, and stillbirth; notably, when cases with vasa previa were excluded, the risks for SGA and stillbirth remained elevated, whereas the increased risk of preterm birth was no longer observed^14^. In our cohort, the incidence of VCI was 0.7%, which is slightly lower than that reported in the literature. Importantly, the VCI group in our study was characterized by higher rates of preterm delivery, primary cesarean section, low Apgar scores, and NICU admissions, further supporting the unfavorable perinatal impact of this placental abnormality.

One potential explanation for the variability in reported outcomes across studies, as well as a limitation of our own analysis, is related to the dynamic nature of placental cord insertions. A recent study demonstrated that cord insertion sites may “migrate” throughout pregnancy, with some marginal insertions resolving to normal positions later in gestation, while a small proportion may progress to velamentous insertion^4^. This phenomenon suggests that the timing of diagnosis and heterogeneity in ultrasound follow-up protocols could partly account for the inconsistent findings in the literature and should be considered when interpreting our results. In addition, the retrospective design of our study and the lack of systematic postnatal placental confirmation of cord insertion represent important limitations and may have influenced the precision of cord insertion classification in some cases. In another institution-based cross-sectional study, high umbilical artery PI values were found to be a reliable predictor of adverse perinatal outcomes in cases with cord insertion anomalies^15^. The absence of Doppler flow assessment in our cohort, therefore, represents an additional limitation of our study.

The strengths of our study include the large sample size, the single-center design, and the standardized evaluation of cord insertion by experienced perinatologists using ISUOG guidelines^8^. Furthermore, marginal and velamentous insertions were analyzed separately, allowing a more precise comparison of outcomes. Another strength is the exclusion of high-risk conditions such as placenta previa, vasa previa, and multiple pregnancies, which helped reduce potential confounding. Limitations include the retrospective design, the absence of Doppler flow assessment, and the potential for cord insertion migration during pregnancy; additionally, the relatively small number of VCI cases may limit the precision of effect estimates and warrants cautious interpretation of subgroup analyses.

## CONCLUSION

The current study shows that MCI is mainly linked to an increased risk of preterm birth. In contrast, VCI is associated with a broader range of adverse perinatal outcomes, including higher rates of preterm birth, low Apgar scores, and NICU admissions. These findings suggest that checking cord insertion during prenatal ultrasound could be an important part of perinatal assessment.

## Data Availability

The datasets generated and/or analyzed during the current study are available from the corresponding author upon reasonable request.
